# Mind the uncertainties – a cross-sectional study on perceptions of cancer risks for factors with inconclusive or limited evidence among the Swedish general public

**DOI:** 10.1186/s12889-026-30362-4

**Published:** 2026-11-13

**Authors:** Ellen Brynskog, Cecilia Hultstrand, Andreas Karlsson Rosenblad, Anna-Lena Sunesson, Thomas Björk Eriksson, Lena Sharp

**Affiliations:** 1 Regional Cancer Centre West, Bergslagsgatan 2, Gothenburg, 411 04 Sweden; 2 Department of health sciences, Karlstad University, Karlstad, Sweden; 3 Regional Cancer Centre North. University hospital of Umeå, Umeå, 901 85 Sweden; 4 Department of nursing, Umeå University, Umeå, 901 87 Sweden; 5 Regional Cancer Centre Stockholm-Gotland, Lindhagensgatan 98, 112 18, Stockholm, 112 18 Sweden; 6 Department of Statistics, Uppsala University, Uppsala, Sweden; 7 Department of Diagnostics and Intervention, Umeå University, Umeå, 901 87 Sweden; 8 Department of Oncology, Institute of Clinical Sciences, Sahlgrenska Academy, University of Gothenburg, Gothenburg, Sweden

**Keywords:** Cancer awareness, Cancer prevention, Cancer misconceptions, Health education, Science communication, JAPreventNCD

## Abstract

**Background:**

Identifying environmental and lifestyle factors that increase the risk of cancer is a complex scientific endeavour, with the degree of certainty regarding carcinogenicity existing along a continuum. To make informed health decisions, the general public must be well informed, both about factors that increase and decrease cancer risk. However, misconceptions can easily gain a foothold in the public consciousness. This study aimed to examine perceptions of carcinogenicity in Sweden regarding factors where the scientific evidence is inconclusive or limited, in relation to demographic strata.

**Methods:**

This cross-sectional study used a pre-existing data set with a randomly selected sample of 1520 participants (18–84 years old) recruited from a Swedish online panel. Statistical analyses utilized post-stratification weights to make the results representative for the general Swedish population. Weighted univariate tests and logistic regression models were used for examining associations between demographic groups and perceptions of carcinogenicity for twelve factors with inconclusive or limited evidence.

**Results:**

Most participants perceived snus (76%), E-cigarettes (67.1%), food additives (63.0%), stress (50.7%), and burnt food (50.4%) as carcinogenic. Whilst no significant differences were observed regarding E-cigarettes, snus was more likely to be perceived as carcinogenic by older (adjusted odds ratio [AOR] 1.01; 95% confidence interval [CI] 1.00–1.02 per year older) and college/university educated (1.47; 1.09–2.00) participants, while it was less likely to be perceived as carcinogenic by males (0.66; 0.48–0.90) and high-income earners (0.62; 0.40–0.95 compared to low-income earners).

Food additives were more often perceived as carcinogenic by older participants (AOR 1.02; 95% CI 1.01–1.03 per year older) and middle-income earners (1.47; 1.03–2.08 compared to low-income earners), while stress and burnt food were more likely to be perceived as carcinogenic by college/university educated (1.49; 1.15–1.91 and 1.54; 1.20–1.98, respectively) participants.

**Conclusions:**

A large proportion of the Swedish general public endorses several unestablished cancer risk factors as carcinogenic. The heterogeneous awareness regarding different factors observed in this study illustrates how challenging it is for individuals to navigate the complex and nuanced scientific evidence regarding cancer risk factors. This highlights the importance of clear and transparent public health messaging.

## Background

The pursuit of identifying agents that are carcinogenic to humans and to what degree, is a broad and complex field of research, engaging scholars from various disciplines, using a range of study formats. The International Agency for Research on Cancer (IARC) and the World Cancer Research Fund (WCRF) are two major actors that continuously aggregate and interpret research findings in this field [1, 2]. The study of carcinogenicity in particular, is shrouded by certain aggravating circumstances, as there is often significant lag time between exposure to a carcinogenic agent and cancer development. Furthermore, experimental manipulation of carcinogenic exposure is generally complex and/or ethically challenging. Evidence of carcinogenicity is often described in terms of degrees of certainty, related to a particular factor or agent.

For example, IARC expert groups review the evidence of carcinogenicity in agents (hazard identification) and categorize them into one of the following five groups, reflecting a decreasing degree of certainty of evidence of carcinogenicity:

- Group 1 (strong evidence of carcinogenicity in humans),
- Group 2 A (probably carcinogenic to humans),
- Group 2B (possibly carcinogenic to humans),
- Group 3 (not classifiable, as evidence is insufficient),
- Group 4 (probably not carcinogenic to humans).

Note that the classification does not indicate the *level* of risk or at what level of exposure risk increases, but rather the strength of the evidence [3]. In a similar but not identical fashion, WCRF grade evidence of whether exposures cause, protects against, or has little effect on cancer risk as either:

- Convincing.
- Probable.
- Substantial effect on risk unlikely.
- Limited–suggestive (evidence is weak, but suggests a direction).
- Limited–no conclusion (the lack of evidence makes any conclusion impossible) [4].

This type of scientific nuance is challenging to communicate to both the general public, decision makers and health care staff in a balanced and understandable manner. The fact that some factors have a strong indirect connection to cancer risk, but evidence of a direct link is absent or insufficient, further aggravates this task. For example, high consumption of certain foods can make it difficult to maintain a healthy body weight which could increase the cancer risk, but the direct link to cancer might be less evident or non-existent. Health literacy, trust in institutions and educational levels are a few of the many factors that play into how scientific communication is both received and perceived [5]. In today’s media landscape, statements from established institutions and legitimate experts are also increasingly competing for attention with actors spreading misleading information and falsehoods, not least on social media platforms [6].

The Swedish general public’s knowledge regarding *established* cancer risk and protective factors varies significantly. Hultstrand et al. [7] found that awareness ranged from 97.1% (smoking) to 9.3% (lack of breastfeeding). These findings are important and provide actionable insights into which risk factors need to be communicated more clearly, as well as how individuals can be better supported to engage in health-promoting behaviours. Equally important however, is the general public’s perceptions regarding factors where evidence of carcinogenicity is lacking or inconclusive, or where carcinogenicity is deemed unlikely. Such insights can help design interventions that combat misinformation and unnecessary worrying amongst the general public. A previous Spanish study surveying journalist undergraduates found that some cancer misconceptions were embraced by a large share of respondents. For example, 32% of the respondents thought that electric power lines cause cancer and 52% agreed that a person’s attitude determines their cancer risk [8]. A Turkish study surveying cancer patients found that 87.6% of respondents thought that genetically modified food causes cancer and 86.6% that stress did [9]. In an Australian study, the corresponding figures were 41% for genetically modified foods and 78% for stress [10].

The current study examines the Swedish general public’s perceptions of the carcinogenicity of the following twelve factors: artificial sweeteners, burnt food, coffee, E-cigarettes, food additives, high-fat diet, milk, mobile phone usage, sleep deprivation, snus (a moist, smokeless tobacco that is common in Sweden), stress and sugar. What these factors have in common is that neither IARC/WCRF nor the scientific community in general position them as (surely) carcinogenic to humans. For coffee and milk, there is instead evidence suggesting cancer protective properties [11, 12]. Below, a brief overview of the current evidence concerning the respective factors included in the study, is provided.

### Artificial sweeteners

Artificial sweeteners are often used in low-calorie products such as soda, chewing gum and flavoured yogurt. Several artificial sweeteners are approved for human consumption, of which *aspartame* is the most studied in relation to cancer risk. Recently, IARC classified aspartame as *possibly carcinogenic to humans* (Group 2b), stating that more research is needed regarding the long-term effects [13]. However, the World Health Organization (WHO) has maintained its recommendation of consuming a maximum of 0–40 mg/kg/day of aspartame, stating that consumption at these levels is safe [14].

### Burnt food

When starchy foods are cooked at high temperatures or until burnt, *acrylamide* is formed. Acrylamide can be found in for example potato chips, potato fries and biscuits [15]. Acrylamide exposure can also come from other sources, such as the plastic industry or tobacco smoke [16]. IARC classify acrylamide as *probably carcinogenic to humans* (Group 2a) [16]. Most of the evidence comes from animal studies, while high-quality human studies are scarce, especially from exposure through food. Food agencies and other institutions recommend cooking foods only to a golden yellow nuance and limiting the consumption of burnt food as a precaution [17–19].

Furthermore, *Polycyclic Aromatic Hydrocarbons* (PAHs) consist of hundreds of compounds formed when organic material is heated or undergoes incomplete combustion, for example through barbecuing. Besides prevalence of PAHs in charred or burnt food, PAHs are also found in for example tobacco smoke and emissions. In animal studies, several PAHs have been shown to increase cancer risk [20]. Benzo[a]pyrene is one of the most studied PAHs and is classified by IARC as *carcinogenic to humans* (Group 1) [21].

### Coffee

WCRF concludes that there is strong evidence that coffee consumption protects against some cancer forms. They also acknowledge that more research is needed to better understand this association [22]. IARC, on the other hand, states that coffee consumption is *not classifiable as to its carcinogenicity to humans* (Group 3), since they view the evidence as inadequate for drawing any conclusions [23].

### E-cigarettes

The European Code Against Cancer (ECAC) [24] includes a recommendation to abstain from use of all forms of tobacco and nicotine-related products, including E-cigarettes. The ECAC concludes that whilst the evidence of the long-term health effects of E-cigarettes (including carcinogenicity) is limited, they generate toxic substances, some of which are known to cause cancer. Additionally, ECAC points out that studies have demonstrated that E-cigarette use increases the likelihood of cigarette smoking initiation, particularly among non-smoking youth [24].

### Food additives

Food additives constitute a heterogeneous group of substances incorporated into food in industrialized processes for technical purposes, such as preservation or enhancement of flavour, colour or texture. Their safety for human health is assessed both nationally and internationally by responsible authorities prior to approval [25].

*Ultra-processed foods* (UPFs), defined as formulations of ingredients produced through multiple industrialized processes, typically contain several classes of food additives [26]. In recent years, numerous studies have examined potential associations between UPF consumption and cancer risk [27–30]. Both the WCRF [31] and the ECAC [24] recommend minimizing UPF intake, as these products often contain high levels of salt, fat and sugar, which can promote weight gain and thereby increase the cancer risk. Obesity and excess body fat in adulthood increases the risk for at least 13 types of cancers [32]. However, concerns have been raised that other components of UPFs might also contribute to an increased cancer risk, including some food additives that have been described as “controversial” [29]. Further research is needed to clarify the role such additives might play in the relationship between UPFs and cancer. However, in the case of *processed meats with added nitrates* such as ham, bacon and salami (considered ultra-processed foods), the evidence of carcinogenicity is convincing. IARC classifies processed meat with added nitrates as *carcinogenic to humans* (Group 1).

### High-fat diet

As stated above, obesity and overweight constitute significant risk factors for cancer [32]. Consequently, the ECAC recommends taking action to avoid or manage overweight and obesity, for example by limiting the consumption of high-fat foods [24]. However, the scientific literature regarding a potential *direct* relationship between fatty foods and cancer is scarce and the findings are inconsistent. For example, dietary fat was associated with risk of breast cancer in pooled analyses combining eight cohorts [33], while a meta-analysis showed that elevated levels of saturated fatty acids was associated with increased risks of breast, prostate and colorectal cancers (although not with lung, pancreatic, ovarian or stomach cancers) [34]. WCRF states that the association between animal fat, fatty acid compositions and cancer is *limited–no conclusion* [12], hence more research is required.

### Milk

WCRF states strong evidence that a diet high in calcium from dairy products, decreases the risk of bowel cancer. However, there is also limited suggestive evidence that dairy consumption increases the risk for prostate cancer. Results regarding other cancer forms are inconclusive [11]. Researchers suggest further investigation to clarify the role of calcium and dairy products in relation to cancer risks [35].

### Mobile phone usage

Since mobile phones emit non-ionising radiation, the question has been raised whether usage increases the risk of brain tumours as the devices are often held near the head of the user. IARC classified radiofrequency electromagnetic fields as *possibly carcinogenic to humans* (Group 2b), concluding that the evidence is limited, in a monograph from 2011 [36]. Since then, a large prospective cohort study has concluded that there is no association between mobile phone usage and the risk of developing glioma, meningioma or acoustic neuroma [37].

### Sleep deprivation

Results from studies investigating the effect of habitual sleep duration and cancer risk are mixed [38–40] and further research is needed to understand the possible associations of too little sleep on cancer risk. Night shift work is more extensively studied and is classified by IARC as *probably carcinogenic to humans* (Group 2a). The pathways in which night shift work increases cancer risk are manifold. One mechanism described by the IARC is that night shift work may hinder the attainment of sufficient sleep, which can impair immune function and alter DNA repair [41].

### Snus

*Snus* (a smokeless, moist tobacco product typically held in the mouth between the lip and upper jaw gums) is common in Sweden, consumed daily by 15% of the adult population in 2024 [42], but used very sparsely in most other countries. Recent studies have shown that snus use does not seem to increase the risk of oral [43] or pancreatic cancer [44]. However, the Swedish Public Health Agency states that evidence regarding this particular tobacco product is insufficient for establishing whether it is carcinogenic to humans [45] The ECAC recommends abstaining from all forms of tobacco and nicotine use [24].

### Stress

A potential association between psychological stress and increased cancer risk has been proposed, although the empirical evidence remains limited and inconsistent [46]. Nevertheless, several biological pathways provide a plausible rationale. Long-term stress elevates circulating levels of cortisol and adrenaline, which can impair immune function and promote systemic inflammation, conditions that may facilitate cancer development [47, 48]. Indirect pathways through unhealthier behaviours in long-term stressed individuals—such as higher alcohol consumption [49] and less physical activity [50]—have also been explored but remain insufficiently understood.

### Sugar

There is strong scientific consensus that diets rich in added sugar, particularly sugar-sweetened beverages, are indirectly linked to increased cancer risk, as such dietary patterns increase the risk of overweight and obesity [22]. For this reason, both the ECAC [24] and the WCRF [51] recommend limiting the consumption of foods and beverages high in sugar. Evidence of a direct effect of sugar consumption has also been investigated, with mechanistic pathways such as increased inflammation and glycaemic load being suggested [52]. However, the research regarding a direct causal association is limited and inconclusive and the pathway through obesity is considered dominant.

### Rationale of this study

Cancer epidemiology is a dynamic and evolving field, in which evidence of carcinogenicity is generated and evaluated through rigorous scientific processes over time. It is vital that the general public trusts these processes and is equipped with evidence-based knowledge regarding cancer risk factors. Previous research from other contexts has found that factors that are evidently non-carcinogenic to humans, or where evidence is weak, have nevertheless gained a foothold in the public consciousness as being carcinogenic [8–10]. It is important to examine such trends in today’s accelerated media landscape, in order to inform the general public about evidence-based actions that can reduce cancer risks, as well as which measures are proven to be less effective. To our knowledge, studies of this kind have previously not been conducted in a Swedish context.

### Aim

This study aimed to examine perceptions of carcinogenicity within the Swedish general public regarding factors for which the scientific evidence does not support classifying the factor as an established human carcinogen, and whether these perceptions vary across demographic strata. A focus of the study was those who actively perceive a given factor as carcinogenic (affirming carcinogenicity).

## Methods

### Study design

The present cross-sectional study is part of a larger study, examining awareness of and attitudes towards the ECAC and cancer risk. A detailed description of the cohort and data collection process is available in a previous publication [53].

### Study population

Study participants were recruited from *Sverigepanelen* (the Sweden panel), a randomly recruited web- based panel with Swedish residents. The panel is organized and operated by Novus, one of Sweden’s leading analysis and research companies. It consists of approximately 50,000 Swedes (18–84 years old), representative of the general population. Notably, since the panel is recruited using simple random sampling, it is not possible to self-register or apply to join the panel. Additionally, the panel is refreshed periodically to limit panel conditioning and attrition effects [54].

### Questionnaire

A study-specific online questionnaire was utilized [53]. The questionnaire consisted of 15 items addressing the perceptions of the carcinogenicity for a range of factors, as well as attitudes, behaviours related to cancer prevention in general and awareness of the ECAC [53]. The factors included in the questionnaire were inspired by the American Institute for Cancer Research 2019 Cancer Risk Awareness Survey [55] and other scholarly work [56, 57]. This was complemented by factors included in the ECAC (version 4, which was the most recently updated version at the time of data collection). Furthermore, four additional factors were included (snus, E-cigarettes, sleep deprivation and milk), based on the authors’ insights regarding currently debated factors in the Swedish context.

### Data collection

Data collection was carried out by Novus during April 2–11, 2024. A total of 3,099 panel members were recruited consecutively until 1,520 participants had completed the questionnaire (response rate 49.0%). The sample size was determined beforehand to allow for reliable subgroup analyses. Once submitted, responses could not be modified. The first 100 responses were reviewed to assess response time, survey completion, item non-response, and the representativeness of respondents relative to the target population, with the aim of identifying potential problems with questionnaire clarity and data quality.

### Study variables

#### Outcomes

A survey statement read “Would you say that the following factors increase the risk of developing cancer?”, followed by a list of factors presented in random order to each participant. For the scope of this study, responses regarding the following twelve factors were analysed: artificial sweeteners, burnt food, coffee, E-cigarettes, food additives, high-fat diet, milk, mobile phone usage, sleep deprivation, snus, stress, and sugar. The response options were “Yes”, “No”, and “Do not know”.

#### Predictors

The demographic characteristics collected from the study sample included the following seven predictors: *gender* (male, female, other), *age* (years), *national background* (Swedish/Foreign), *education level* (highest completed: primary school, secondary school, or college/university), *income* (individual income measured in Swedish Krona (SEK; €1 ≈ 10 SEK) per month, categorized into three groups: < 20,000 SEK/month or Other, 20,000–39,999 SEK/month, and ≥ 40,000 SEK/month, with those responding “No income” classified as < 20,000 SEK/month and “Don’t know” or “Does not want to disclose” classified as Other), *living alone* (Yes/No), and *Swedish Health Care Regions* (HCRs; six regions: Stockholm-Gotland, Mid-Sweden, Southeast, South, West, and North). *Living alone* was constructed by dichotomizing a variable measuring marital status (married, cohabiting, living alone, partnership, living with parents, and other), with those providing their marital status as “living alone” classified as Yes and all the other classified as No. Moreover, since no respondent answered “other” for gender, it was dichotomized as male/female. The inclusion of Swedish HCRs as predictors should be considered exploratory since these are purely administrative areas and there are no strong theoretical justifications for expecting to observe differences between these.

### Statistical analyses

Categorical data are presented as frequencies and percentages, n (%), while continuous data are provided as mean values with accompanying standard deviations (SDs). In the questionnaire, the participants were asked the question “Would you say that the following factors increase the risk of developing cancer?”, followed by a list with the twelve studied factors. For each question, the three response options were “Yes”, “No”, and “Do not know”. Since the focus of the present study was those who actively perceive a given factor as carcinogenic (*affirming carcinogenicity*), with no intention of trying to distinguish uncertainty from rejection, these answers were in the statistical analyses dichotomized as Yes/No, with “Do not know” responses included in the “No” category. Collapsing “Do not know” with “No” operationalises *not affirming carcinogenicity*, which is the relevant contrast for our research question. Unless otherwise specified, the statistical analyses were performed using the sample post-stratification weights, to make the results representative for the general Swedish population. Tests of differences between demographic groups were performed using Pearson’s χ^2^-test utilizing Rao-Scott second-order corrections, with P-values obtained from a Satterthwaite approximation of the distribution using denominator degrees of freedom as described by Thomas and Rao [58].

To estimate the magnitude of the association between demographic characteristics and the perception of the twelve studied factors (as being risk factors for cancer), weighted adjusted logistic regression models were calculated separately for the twelve risk factors using generalised linear models with a quasi-binomial family and a logit link function, together with inverse-probability weighting and design-based standard errors. For all models, the predicted outcome of interest was “Yes” (i.e., the respondent considered the factor as a cancer risk), with all predictors included simultaneously as independent variables. Age (years) was included as a continuous variable, Female was used as reference category for gender, Foreign (including missing values) as reference category for national background, “No” as reference category for college/university education, “< 20,000 / Other” as reference category for income, “No” as reference category for living alone, and Stockholm-Gotland as reference category for HCR. The results are reported as adjusted odds ratios (AORs) with 95% confidence intervals (CIs).

Statistical analyses were performed using R 4.3.1 or later (R Foundation for Statistical Computing, Vienna, Austria) with the R package survey [58] used to handle the sampling-weighted analyses. Two-sided P-values < 0.05 were considered statistically significant. No adjustment for multiplicity was applied since such adjustments rest on the premise of a universal null hypothesis, i.e., that all associations examined are null, which is not a reasonable premise for associations between demographic characteristics and health beliefs [59]. Each estimate should be interpreted on its own merits, with emphasis on the magnitude and precision of the AORs and their 95% CIs.

## Results

Table 1 summarizes the weighted and unweighted demographic characteristics of the 1520 respondents. A slight male predominance was observed (50.5% male, 49.5% female). Participants were older than the general population, with an unweighted mean (SD) age of 53.3 (17.5) years, which decreased to 48.7 (18.5) years after weighting. Adults aged 18–34 were underrepresented (18.9%) and up-weighted to 28.1%, whereas those aged 65–84 were overrepresented (30.5%) and down-weighted to 23.4%. Individuals with a Swedish background were slightly overrepresented (81.8%) and down-weighted to 80.0%.

**Table 1:** Demographic characteristics (weighted and unweighted distributions) for the 1520 participants

Variable	Unweighted		Weighted	
	n	%	n	%
Gender				
Male	767	50.5	768	50.5
Female	753	49.5	752	49.5
Age (years)^a^				
18–34	288	18.9	428	28.1
35–49	362	23.8	376	24.7
50–64	407	26.8	361	23.8
65–84	463	30.5	355	23.4
National background				
Swedish	1243	81.8	1216	80.0
Foreign	102	6.7	99	6.5
Missing	175	11.5	205	13.5
Education level				
Primary school	65	4.3	114	7.5
Secondary school	506	33.3	812	53.4
College / University	949	62.4	594	39.1
Income (SEK/month)				
< 20,000	323	21.2	427	28.1
20,000–39,999	595	39.1	598	39.3
≥ 40,000	538	35.4	433	28.5
Don’t know / Does not want to disclose	64	4.2	62	4.1
Living alone				
Yes	366	24.1	378	24.8
No	1154	75.9	1142	75.2
Swedish Health Care Region				
Stockholm-Gotland	393	25.9	373.1	24.5
Mid-Sweden	312	20.5	314.6	20.7
Southeast	135	8.9	148.8	9.8
South	253	16.6	277.1	18.2
West	274	18.0	274.4	18.1
North	153	10.1	131.9	8.7

*SD* standard deviation, *SEK* Swedish Krona. 10,000 SEK ≈ €885

^a^ The unweighted mean (SD) age was 53.3 (17.5) years, while the weighted mean (SD) age was 48.7 (18.5) years

Participants with a college or university education were strongly overrepresented (62.4%) and down-weighted to 39.1%, while those with a secondary school education were up-weighted from 33.3% to 53.4%. Lower-income participants (< 20,000 SEK/month) were up-weighted from 21.2% to 28.1%, whereas higher-income participants (≥ 40,000 SEK/month) were down-weighted from 35.4% to 28.5%. Living alone was largely representative (24.1%) and required minimal adjustment (24.8%), and geographic region of residence (Swedish HCRs) was also broadly representative, with only minor weighting adjustments.

### Perceived carcinogenicity of factors

**Fig. 1:**
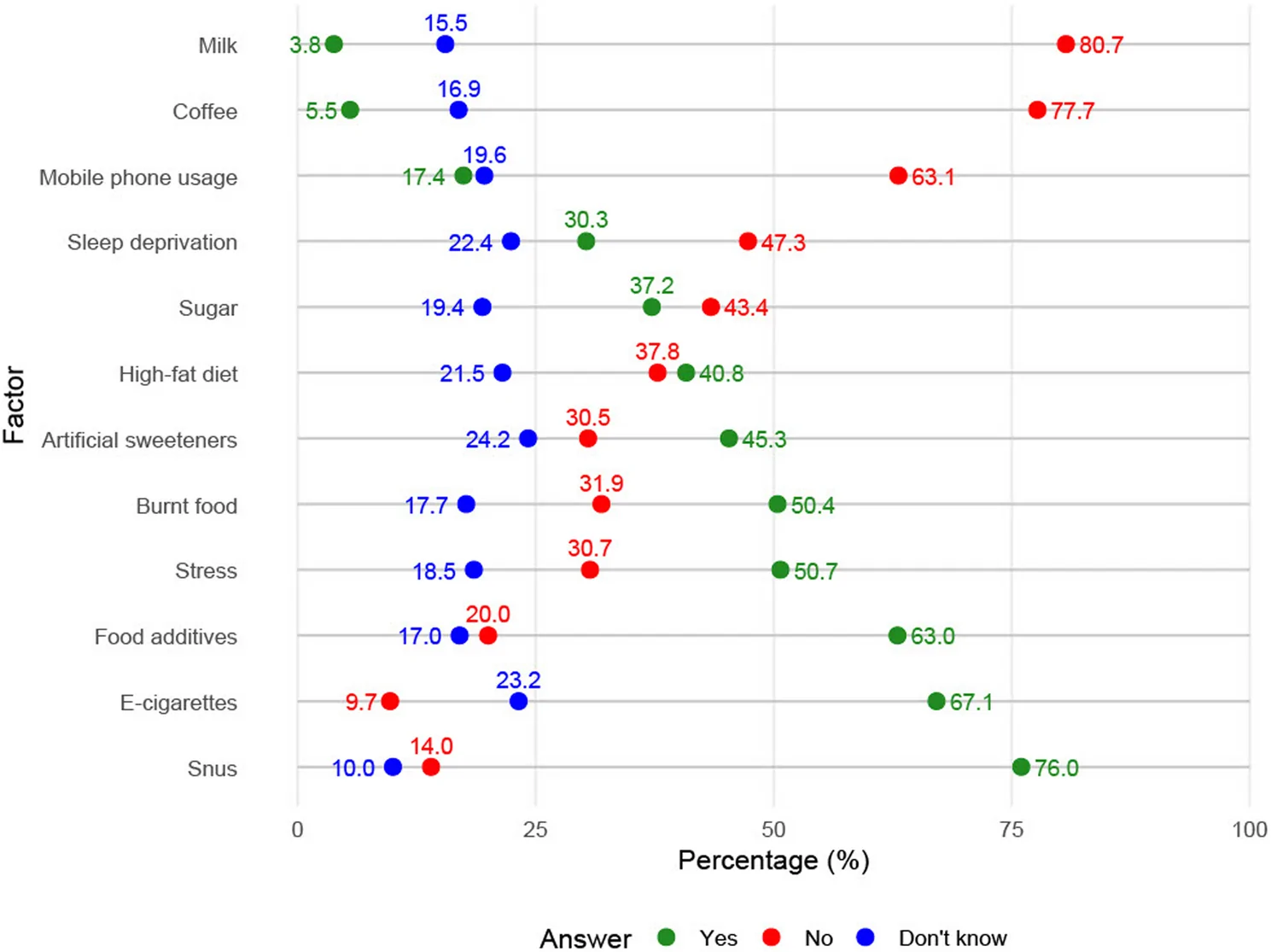
Participants perceptions of carcinogenicity for the twelve factors, as a response to the question “Would you say that the following factors increase the risk of developing cancer?”

Weighted distributions of the three response options “Yes”, “No”, and “Don’t know” as answers to the question “Would you say that the following factors increase the risk of developing cancer?” for the twelve factors included in the present study are provided in Fig. 1. 

*Milk* and *coffee* were thought of as carcinogenic by only 3.8% and 5.5% of the respondents, respectively. Conversely *snus*, was the factor that the largest proportion of respondents perceived as carcinogenic (76.0%). Besides *snus*, a majority of the participants also perceived *burnt food* (50.4%), *stress* (50.7%), *food additives* (63.0%), and *E-cigarettes* (67.1%) as carcinogenic. *Artificial sweeteners* and *E-cigarettes* at 24.2% and 23.2% respectively, were in turn the factors for which the largest proportion of participants responded, “Don’t know”.

### Perceived carcinogenicity of factors across demographic groups

The unadjusted perceived carcinogenicities of the twelve factors, according to the weighted distribution of the demographic characteristics, are provided in Tables 2, 3 and 4. Notably, we found no significant differences in perceived carcinogenicity for any demographic characteristic regarding *artificial sweeteners*, *E-cigarettes*, and *sugar*.

**Table 2:** Perception of Artificial sweeteners, Burnt food, Coffee, and E-cigarettes as risk factors for cancer according to the weighted distribution of the demographic characteristics

Variable	Artificial sweeteners			Burnt food			Coffee			E-cigarettes		
	*n*	%	*P*-value	*n*	%	*P*-value	*n*	%	*P*-value	*n*	%	*P*-value
Total	689	45.3	N/A	766	50.4	N/A	83	5.5	N/A	1020	67.1	N/A
Gender			0.642			0.228			0.744			0.878
Male	354	46.1		372	48.5		40	5.2		517	67.3	
Female	335	44.5		394	52.4		43	5.7		503	66.9	
Age (years)			0.692			0.156			**0.031**			0.332
18–34	187	43.9		207	48.3		36	8.5		292	68.4	
35–49	177	47.2		180	47.8		23	6.2		266	70.8	
50–64	171	47.3		177	49.0		13	3.6		235	65.1	
65–84	153	43.1		202	57.0		10	2.9		226	63.6	
National background			0.156			0.788			**< 0.001**			0.195
Swedish	536	44.1		610	50.2		46	3.8		804	66.1	
Foreign / Missing	153	50.2		156	51.3		37	12.0		216	71.1	
College / University education			0.321			**0.002**			0.603			0.839
Yes	258	43.5		334	56.2		35	6.0		401	67.4	
No	430	46.5		432	46.7		47	5.1		619	66.9	
Income (SEK/month)			0.147			0.524			0.639			0.321
< 20,000 / Other^a^	205	42.0		256	52.3		30	6.2		317	64.9	
20,000–39,999	294	49.1		288	48.2		34	5.6		418	69.9	
≥ 40,000	190	43.8		222	51.2		19	4.3		285	65.8	
Living alone			0.568			0.745			0.993			0.309
Yes	165	43.7		194	51.3		21	5.5		243	64.4	
No	524	45.8		572	50.1		62	5.4		777	68.0	
Swedish Health Care Region			0.379			0.273			0.805			0.510
Stockholm-Gotland	153	41.1		186	50.0		17	4.6		260	69.6	
Mid-Sweden	134	42.5		143	45.5		16	5.0		211	67.2	
Southeast	67	45.1		83	55.9		10	6.8		99	66.6	
South	131	47.4		140	50.5		20	7.4		173	62.4	
West	133	48.5		154	56.1		12	4.5		179	65.4	
North	70	53.0		59	44.9		7	5.6		97	73.8	

*N/A* not applicable, *SEK* Swedish Krona (10,000 SEK ≈ €885). Frequencies and percentages are estimated based on post-stratification weights and then rounded, meaning that not all numbers may add up due to rounding errors. P-values are calculated using Pearson’s χ^2^-statistic with the Rao-Scott second-order correction. Significant *P*-values are given in **bold**. ^a^ Including *Don’t know* and *Does not want to disclose*

**Table 3:** Perception of Food additives, High-fat diet, Milk, and Mobile phone usage as risk factors for cancer according to the weighted distribution of the demographic characteristics

Variable	Food additives			High-fat diet			Milk			Mobile phone usage		
	*n*	%	*P*-value	*n*	%	*P*-value	*n*	%	*P*-value	*n*	%	*P*-value
Total	958	63.0	N/A	619	40.8	N/A	58	3.8	N/A	264	17.4	N/A
Gender			0.71			0.101			0.809			**0.034**
Male	488	63.6		293	38.2		31	4.0		113	14.7	
Female	469	62.4		326	43.4		28	3.7		151	20.1	
Age (years)			**< 0.001**			**0.018**			0.051			**0.018**
18–34	221	51.7		155	36.2		27	6.4		78	18.3	
35–49	229	60.9		139	37.0		12	3.1		84	22.5	
50–64	256	70.8		152	42.0		12	3.4		62	17.3	
65–84	252	71.0		174	48.9		7	1.9		39	10.9	
National background			0.386			0.888			**< 0.001**			0.518
Swedish	757	62.3		494	40.6		32	2.6		206	16.9	
Foreign / Missing	200	66.0		125	41.2		26	8.7		58	19.2	
College / University education			0.378			0.559			0.593			0.077
Yes	384	64.6		248	41.8		25	4.3		88	14.9	
No	574	62.0		371	40.1		33	3.6		175	19.0	
Income (SEK/month)			**0.007**			0.144			0.135			0.484
< 20,000 / Other^a^	283	57.9		197	40.4		27	5.6		84	17.2	
20,000–39,999	414	69.2		264	44.1		15	2.6		114	19.1	
≥ 40,000	261	60.3		158	36.6		15	3.6		66	15.3	
Living alone			0.691			0.247			0.214			0.753
Yes	234	61.9		142	37.7		20	5.3		63	16.7	
No	724	63.4		477	41.8		38	3.3		201	17.6	
Swedish Health Care Region			0.889			0.780			0.262			0.477
Stockholm-Gotland	232	62.1		159	42.7		18	4.8		59	15.8	
Mid-Sweden	190	60.5		118	37.5		7	2.2		58	18.5	
Southeast	100	67.3		67	44.8		1	0.9		33	22.3	
South	174	62.8		106	38.2		9	3.4		37	13.4	
West	175	63.7		115	41.9		17	6.1		48	17.5	
North	87	66.1		54	41.2		5	4.1		28	21.4	

*N/A* not applicable, *SEK* Swedish Krona (10,000 SEK ≈ €885). Frequencies and percentages are estimated based on post-stratification weights and then rounded, meaning that not all numbers may add up due to rounding errors. *P*-values are calculated using Pearson’s χ^2^-statistic with the Rao-Scott second-order correction. Significant P-values are given in **bold**. ^a^ Including *Don’t know* and *Does not want to disclose*

**Table 4:** Perception of Sleep deprivation, Snus, Stress, and Sugar as risk factors for cancer according to the weighted distribution of the demographic characteristics

Variable	Sleep deprivation			Snus			Stress			Sugar		
	*n*	%	*P*-value	*n*	%	*P*-value	*n*	%	*P*-value	*n*	%	*P*-value
Total	461	30.3	N/A	1156	76.0	N/A	771	50.7	N/A	566	37.2	N/A
Gender			0.073			**< 0.001**			0.760			0.595
Male	253	33.0		547	71.2		386	50.2		280	36.4	
Female	208	27.6		609	81.0		385	51.2		286	38.1	
Age (years)			**< 0.001**			0.183			**0.019**			0.094
18–34	163	38.1		314	73.5		250	58.4		175	40.9	
35–49	125	33.2		277	73.8		183	48.7		154	40.9	
50–64	90	24.9		277	76.6		162	44.8		120	33.2	
65–84	83	23.5		288	81.0		176	49.7		117	33.0	
National background			0.184			0.903			**0.045**			0.430
Swedish	356	29.3		926	76.1		596	49.0		445	36.6	
Foreign / Missing	105	34.6		230	75.7		175	57.7		121	39.9	
College / University education			**0.048**			**0.033**			**0.022**			0.250
Yes	201	33.8		471	79.3		326	54.9		233	39.3	
No	260	28.1		685	74.0		445	48.0		332	35.9	
Income (SEK/month)			0.938			0.190			0.456			0.153
< 20,000 / Other^a^	147	30.1		387	79.1		261	53.4		161	32.8	
20,000–39,999	179	29.9		454	76.0		301	50.4		239	40.0	
≥ 40,000	135	31.1		315	72.6		209	48.3		166	38.4	
Living alone			0.744			0.127			0.308			0.989
Yes	111	29.5		273	72.4		202	53.6		141	37.3	
No	350	30.6		882	77.2		569	49.8		425	37.2	
Swedish Health Care Region			0.639			0.107			0.458			0.547
Stockholm-Gotland	102	27.4		276	73.8		188	50.5		136	36.4	
Mid-Sweden	86	27.2		224	71.2		142	45.3		111	35.3	
Southeast	50	33.4		122	82.0		86	57.9		67	45.1	
South	92	33.2		205	74.1		141	50.8		95	34.3	
West	87	31.8		223	81.4		143	52.2		102	37.2	
North	44	33.3		106	80.0		70	53.1		55	41.4	

*N/A* not applicable, *SEK* Swedish Krona (10,000 SEK ≈ €885). Frequencies and percentages are estimated based on post-stratification weights and then rounded, meaning that not all numbers may add up due to rounding errors. *P*-values are calculated using Pearson’s χ^2^-statistic with the Rao-Scott second-order correction. Significant *P*-values are given in **bold**. ^a^ Including *Don’t know* and *Does not want to disclose*

A significant difference between age groups regarding perceived carcinogenicity was observed for half of the factors (*coffee*, *food additives*, *high-fat diet*, *mobile phone usage*, *sleep deprivation*, and *stress)* while a significant difference between those with and without a college/university education was observed for four factors (*burnt food*, *sleep deprivation*, *snus*, and *stress*). It was more common among respondents with a college/university education to perceive these factors as carcinogenic. For national background, significant differences in perceived carcinogenicity were noted for three of the factors (*coffee*, *milk*, and *stress)*, with those having a Swedish background in all cases less often perceiving these as carcinogenic. A significant difference between males and females was observed for *mobile phone usage* and *snus*, with women in both cases more often tending to perceive these as carcinogenic. A significant difference in perceived carcinogenicity between income levels was observed only for *food additives*. Notably, we found no significant differences among respondents living alone or between HCRs/geographic areas in perceived carcinogenicity for any of the factors.

### Associations between demographic characteristics and perceived carcinogenicity of factors

Results from weighted adjusted logistic regression analyses of the association between demographic characteristics and perceived carcinogenicity of the twelve factors are provided in Tables 5, 6 and 7. No demographic characteristic was significantly associated with perceiving *E-cigarettes* as carcinogenic. For *artificial sweeteners*, a significant association with perceived carcinogenicity was observed for those living in the northern HCR, with a 1.64 times higher chance of perceiving them as carcinogenic compared with those living in the Stockholm-Gotland HCR (*P* = 0.035). No other variable turned out to be statistically significant for this factor. This was also the only factor for which HCR showed a significant association.

**Table 5:** Results from weighted adjusted logistic regression analyses of the association between demographic characteristics and perception of Artificial sweeteners, Burnt food, Coffee, and E-cigarettes as risk factors for cancer

Variable	Artificial sweeteners			Burnt food			Coffee			E-cigarettes		
	AOR	95% CI	*P*-value	AOR	95% CI	*P*-value	AOR	95% CI	*P*-value	AOR	95% CI	*P*-value
Male gender	1.03	0.79–1.35	0.805	0.94	0.71–1.23	0.633	0.97	0.52–1.83	0.936	1.03	0.77–1.36	0.862
Age (years)	1.00	0.99–1.01	0.854	1.01	1.00-1.01	0.062	0.98	0.97-1.00	**0.014**	0.99	0.99-1.00	0.200
Swedish background	0.78	0.55–1.09	0.143	0.91	0.65–1.28	0.592	0.31	0.16–0.60	**0.001**	0.81	0.56–1.16	0.241
College / University education	0.87	0.68–1.12	0.279	1.54	1.20–1.98	**0.001**	1.42	0.75–2.71	0.285	1.04	0.79–1.35	0.800
Income (SEK/month)												
< 20,000 / Other^a^	Ref.			Ref.			Ref.			Ref.		
20,000–39,999	1.38	0.99–1.92	0.054	0.79	0.57–1.11	0.172	0.99	0.48–2.06	0.978	1.26	0.89–1.77	0.193
≥ 40,000	1.16	0.82–1.66	0.402	0.80	0.56–1.14	0.221	0.74	0.31–1.73	0.481	1.00	0.69–1.46	0.990
Living alone	0.94	0.70–1.26	0.681	1.03	0.76–1.38	0.859	1.08	0.52–2.27	0.831	0.86	0.63–1.17	0.332
Swedish Health Care Region												
Stockholm-Gotland	Ref.			Ref.			Ref.			Ref.		
Mid-Sweden	1.05	0.72–1.53	0.815	0.87	0.60–1.26	0.453	1.11	0.43–2.83	0.834	0.88	0.59–1.30	0.516
Southeast	1.20	0.73–1.97	0.466	1.36	0.83–2.24	0.225	1.76	0.66–4.71	0.261	0.87	0.52–1.48	0.618
South	1.28	0.86–1.92	0.228	1.05	0.69–1.58	0.828	1.54	0.58–4.07	0.387	0.71	0.47–1.09	0.116
West	1.37	0.93–2.01	0.106	1.29	0.87–1.90	0.205	0.96	0.38–2.41	0.934	0.83	0.55–1.24	0.350
North	1.64	1.04–2.59	**0.035**	0.86	0.54–1.35	0.506	1.16	0.43–3.10	0.767	1.20	0.71–2.03	0.504

*AOR* adjusted odds ratio, *CI* confidence interval, *Ref*. reference category, *SEK* Swedish Krona (10,000 SEK ≈ €885). The regression models included all variables in the table. Significant P-values are given in **bold**. ^a^ Including *Don’t know* and *Does not want to disclose*

**Table 6:** Results from weighted adjusted logistic regression analyses of the association between demographic characteristics and perception of Food additives, High-fat diet, Milk, and Mobile phone usage as risk factors for cancer

Variable	Food additives			High-fat diet			Milk			Mobile phone usage		
	AOR	95% CI	*P*-value	AOR	95% CI	*P*-value	AOR	95% CI	*P*-value	AOR	95% CI	*P*-value
Male gender	1.12	0.84–1.49	0.433	0.86	0.65–1.13	0.268	1.21	0.57–2.56	0.617	0.65	0.45–0.93	**0.017**
Age (years)	1.02	1.01–1.03	**< 0.001**	1.01	1.00-1.02	**0.006**	0.98	0.96-1.00	0.112	0.99	0.98-1.00	**0.035**
Swedish background	0.74	0.51–1.07	0.107	0.92	0.65–1.30	0.636	0.31	0.14–0.69	**0.004**	0.87	0.55–1.39	0.568
College / University education	1.16	0.89–1.51	0.284	1.12	0.87–1.43	0.385	1.46	0.74–2.87	0.275	0.70	0.48–1.02	0.066
Income (SEK/month)												
< 20,000 / Other^a^	Ref.			Ref.			Ref.			Ref.		
20,000–39,999	1.47	1.03–2.08	**0.032**	1.11	0.79–1.56	0.535	0.48	0.23–1.02	0.057	1.33	0.85–2.07	0.208
≥ 40,000	0.93	0.65–1.34	0.703	0.79	0.55–1.12	0.189	0.57	0.27–1.24	0.158	1.22	0.74–2.01	0.441
Living alone	0.89	0.65–1.21	0.462	0.79	0.59–1.06	0.122	1.72	0.80–3.72	0.166	0.98	0.65–1.49	0.941
Swedish Health Care Region												
Stockholm-Gotland	Ref.			Ref.			Ref.			Ref.		
Mid-Sweden	0.93	0.63–1.38	0.726	0.79	0.54–1.16	0.222	0.48	0.18–1.26	0.133	1.17	0.68–1.99	0.569
Southeast	1.38	0.81–2.35	0.233	1.09	0.66–1.78	0.745	0.22	0.04–1.16	0.075	1.45	0.75–2.81	0.274
South	1.03	0.67–1.59	0.887	0.81	0.54–1.24	0.333	0.61	0.17–2.27	0.465	0.81	0.44–1.49	0.496
West	1.10	0.73–1.66	0.652	0.94	0.63–1.40	0.774	1.29	0.51–3.27	0.587	1.11	0.65–1.90	0.700
North	1.36	0.83–2.24	0.221	0.97	0.60–1.56	0.905	0.81	0.29–2.25	0.685	1.35	0.73–2.50	0.342

*AOR* adjusted odds ratio, *CI* confidence interval, *Ref.* reference category, *SEK* Swedish Krona (10,000 SEK ≈ €885). The regression models included all variables in the table. Significant P-values are given in **bold**. ^a^ Including *Don’t know* and *Does not want to disclose*

**Table 7:** Results from weighted adjusted logistic regression analyses of the association between demographic characteristics and perception of Sleep deprivation, Snus, Stress, and Sugar as risk factors for cancer

Variable	Sleep deprivation			Snus			Stress			Sugar		
	AOR	95% CI	*P*-value	AOR	95% CI	*P*-value	AOR	95% CI	*P*-value	AOR	95% CI	*P*-value
Male gender	1.36	1.01–1.82	**0.042**	0.66	0.48–0.90	**0.008**	1.06	0.81–1.39	0.655	0.90	0.68–1.19	0.473
Age (years)	0.98	0.98–0.99	**< 0.001**	1.01	1.00-1.02	**0.021**	0.99	0.99-1.00	0.101	0.99	0.99-1.00	0.066
Swedish background	0.84	0.58–1.21	0.345	0.97	0.65–1.45	0.892	0.72	0.51–1.01	0.055	0.87	0.61–1.24	0.448
College / University education	1.45	1.10–1.91	**0.009**	1.47	1.09-2.00	**0.012**	1.49	1.15–1.91	**0.002**	1.10	0.85–1.42	0.484
Income (SEK/month)												
< 20,000 / Other^a^	Ref.			Ref.			Ref.			Ref.		
20,000–39,999	1.00	0.70–1.44	0.996	0.79	0.52–1.18	0.249	0.88	0.63–1.22	0.431	1.43	1.01–2.04	**0.044**
≥ 40,000	0.92	0.63–1.35	0.667	0.62	0.40–0.95	**0.029**	0.72	0.51–1.02	0.068	1.33	0.92–1.93	0.125
Living alone	1.00	0.72–1.38	0.991	0.72	0.52–1.01	0.056	1.17	0.87–1.56	0.307	1.06	0.78–1.43	0.713
Swedish Health Care Region												
Stockholm-Gotland	Ref.			Ref.			Ref.			Ref.		
Mid-Sweden	1.00	0.67–1.51	0.982	0.86	0.56–1.32	0.495	0.82	0.56–1.19	0.299	0.97	0.65–1.43	0.861
Southeast	1.39	0.82–2.36	0.219	1.61	0.88–2.92	0.119	1.42	0.87–2.32	0.163	1.50	0.91–2.47	0.109
South	1.32	0.84–2.08	0.224	1.00	0.63–1.59	0.995	1.00	0.66–1.51	0.997	0.94	0.61–1.44	0.766
West	1.27	0.83–1.93	0.270	1.47	0.92–2.35	0.106	1.08	0.74–1.57	0.703	1.07	0.72–1.59	0.743
North	1.24	0.75–2.04	0.399	1.48	0.83–2.63	0.183	1.07	0.67–1.72	0.775	1.22	0.76–1.95	0.421

*AOR* adjusted odds ratio, *CI* confidence interval, *Ref.* reference category, *SEK* Swedish Krona (10,000 SEK ≈ €885). The regression models included all variables in the table. Significant P-values are given in **bold**. ^a^ Including *Don’t know* and *Does not want to disclose*

Having a college/university education implied a 1.54 times higher chance of perceiving *burnt food* as carcinogenic (*P* = 0.001) and was the only variable showing a statistically significant association with this factor. In addition, having a college/university education was associated with a 1.45 times higher chance of perceiving *sleep deprivation* as carcinogenic (*P* = 0.009), a 1.47 times higher chance of perceiving *snus* as carcinogenic (*P* = 0.012), and a 1.49 times higher chance of perceiving *stress* as carcinogenic (*P* = 0.002), compared to those having no college/university education. Notably, college/university education was the only demographic characteristic that was significantly associated with perceiving *stress* as carcinogenic.

Age was significantly associated with perceiving *coffee* as a carcinogenic factor, with increasing age implying a lower chance (AOR 0.98 for each additional year of age; *P* = 0.014). Increased age was also significantly associated with lower chances of perceiving *mobile phone usage* (AOR 0.99 for each additional year of age; *P* = 0.035) and *sleep deprivation* (AOR 0.98 for each additional year of age; *P* < 0.001) as carcinogenic. Conversely, each additional year of age was associated with a 1.02 times higher chance of perceiving *food additives* as carcinogenic (*P* < 0.001), a 1.01 times higher chance of perceiving a *high-fat diet* as carcinogenic (*P* = 0.006), and a 1.01 times higher chance of perceiving *snus* as carcinogenic (*P* = 0.021). Notably, age was the only demographic characteristic that was significantly associated with perceiving a high-fat diet as carcinogenic.

National background was significantly associated with perceiving *coffee* (AOR 0.31; *P* = 0.001) and *milk* (AOR 0.31; *P* = 0.004) as carcinogenic, with a Swedish background implying a lower chance of this perception. Additionally, national background was the only demographic characteristic that was significantly associated with perceiving *milk* as carcinogenic.

Gender was significantly associated with perceptions on carcinogenicity of *mobile phone usage*, *sleep deprivation*, and *snus* usage. Males were thus 1.36 times more likely to perceive sleep deprivation as carcinogenic (*P* = 0.042) but were less likely to perceive *mobile phone usage* (AOR 0.65; *P* = 0.017) or *snus* (AOR 0.66; *P* = 0.008) as carcinogenic.

Finally, income level was significantly associated with perceiving *food additives*, *snus*, and *sugar* as carcinogenic. Those in the middle-income bracket (SEK/month 20,000–39,999) were 1.47 times more likely to perceive *food additives* (*P* = 0.032) and 1.43 times more likely to perceive *sugar* as carcinogenic (*P* = 0.044), compared to those in the lowest income bracket (SEK/month < 20,000 /Other). Conversely, those in the upper income bracket (SEK/month ≥ 40,000) were less likely (compared to respondents in the lowest income bracket) to perceive *snus* as carcinogenic (AOR 0.62; *P* = 0.029). Notably, income level was the only demographic characteristic that was significantly associated with perceiving *sugar* as carcinogenic.

## Discussion

This cross-sectional study shows how the Swedish general public perceive the carcinogenicity of a range of factors, for which evidence is limited or inconclusive. A majority of participants perceived *snus* (76%), *E-cigarettes* (67.1%), *food additives* (63.0%), *stress* (50.7%), and *burnt food* (50.4%) as carcinogenic.

### Results in context

A larger share of the respondents in the current study perceived *artificial sweeteners* as carcinogenic (45.3%), compared to 14.7% in a Turkish study [9], but similar to the findings of a Spanish study (40.2%) [8]. A notable finding was that a minority (17.4%) in our sample perceived *mobile phone usage* as carcinogenic, compared to 77.8% and 48.2% in the Turkish and Spanish studies, respectively. In a similar Australian study, the corresponding figure was 59.8% [10]. We can only speculate as to why the findings deviate. Possibly, these differences can be attributed to the varying population groups that were sampled in the respective studies; Dominguez and Sapiña [8] surveyed university students, whilst Uzun and Akin [9] surveyed patients admitted to a hospital. The data set of MacTiernan et al. [10] is relatively old (data collected in 2007–2008), which might also explain these differences.

In a previous publication on established cancer risk factors, we found a relatively clear-cut educational gradient in the data, where people with higher education to a greater extent were aware of cancer-causing factors in that they provided the “correct answer” more often on a group level [7]. However, in the present study, we found no distinct gradient for either education, income, or country of origin. This indicates that misconceptions and uncertainties are widespread, affecting most demographic groups. Furthermore, in the present study the “correct answer” is often unclear. Interpretation of the scientific grading is much less straightforward as compared to other factors that are considered established cancer risk factors. The study results can be seen as a testament of how difficult it is for individuals in today’s society to assess and evaluate sound, yet complex scientific evidence on cancer risk factors.

We did find some statistically significant differences across age groups in univariate analyses, where younger people were more likely to perceive *coffee*, *mobile phone usage*, *sleep deprivation*, and *stress* to cause cancer, whilst the older age groups were more likely to perceive *food additives* as carcinogenic. In this context, the significant shift that has taken place in recent decades regarding how the general public receives health information is noteworthy and might possibly play a part in the age differences seen in our results. Traditional media has largely been replaced by social media platforms and artificial intelligence (AI), as people’s daily source of information and news [60]. Social media enables messages to be altered and adapted as they are shared among users, and visibility of content is heavily shaped by algorithms that reward fast interaction. This contributes to rapid dissemination of inaccurate information. In fact, research shows that inaccurate information spreads faster, deeper and broader compared to accurate information online [61]. This relatively new and speedily developing way of communication poses a significant challenge that the scientific and public health community must adapt to [6].

Risk communication is a complex endeavour, especially regarding factors such as those covered in the current study, where the scientific evidence is limited or inconclusive, or where the dominant pathway is an indirect link to increased cancer risk. An additional complexity highlighted by this study concerns the interpretation of *“No”* versus *“Do not know”* responses, and the implications of this distinction for public health communication. Misconceptions and lack of knowledge can be viewed as different positions along a continuum of understanding, potentially requiring different communication strategies. Addressing firmly held but inaccurate beliefs may necessitate different interventions than addressing uncertainty or limited knowledge about potential cancer risk factors. The scientific literature on risk communication highlights the importance of transparent and accurate risk reporting, considering social and individual context, to minimize the risk of stigmatization and blame [62]. Plain language, visual aids, and communicating absolute rather than relative risks have also been suggested [63].

### Strengths and limitations

The strengths of this study include the large, representative, weighted sample as well as the fact that this topic is previously unstudied in a Swedish context. In hindsight, including some additional factors often discussed in relation to cancer in recent years, such as UPFs and dietary supplements, could have strengthened the study. A limitation with the questionnaire used in the study was that it failed to include items investigating how strongly the participants felt regarding their responses and also if any actions had been taken to reduce exposure to certain factors (as a way to reduce the cancer risk). Moreover, the twelve factors analysed in the present study were mixed with established, well-known cancer risk factors, such as cigarette smoking, UV-radiation, and obesity. It is possible that the mix of items might have introduced a framing effect bias [64], where participants become more prone to believing that all factors in the survey were related to cancer. No cognitive interviews were conducted prior to data collection initiation.

A limitation of outsourcing the recruitment and handling of the survey was that the authors had no access to data on non-responders, thus precluding any sensitivity analyses. Additionally, although almost all in Sweden have internet access [65], it should be acknowledged that the small groups without internet access, including some elderly individuals and those with lower digital literacy, are structurally excluded from the sampling frame used for the study. Given that these groups may hold systematically different cancer risk perceptions, the generalizability of our findings to these sub-populations is uncertain.

Although a significant effect of HCRs on artificial sweeteners was found, it should be noted that the inclusion of HCRs as predictors should be considered exploratory since these are purely administrative areas and there are no strong theoretical justifications for why these should differ.

Finally, it should be noted that while we measured and adjusted for several important characteristics such as sex, age, education level, income, and national background, there were other possible important lifestyle factors that were not measured, such as e.g. smoking and alcohol consumption, as well as experiences of previous or current cancer diagnoses among the responders. This makes it possible that some residual confounding remained and could have affected the results. Additionally, while weighting may improve alignment with population demographics, there could still remain potential selection biases associated with participation.

### Implications for practice and future research

Some communicators may utilize the term *myths* to describe unestablished risk factors, or state that respondents are *incorrect* to believe that different factors cause cancer. Although this terminology is understandable, a more nuanced and empathic tone is necessary in this conversation. Communicators as well as researchers must remain humble to the fact that this scientific field is both dynamic and continuously evolving. As outlined in the background, there are substantial uncertainties within the existing evidence base regarding the factors included in this study. Moreover, if the scientific community cannot fully determine what is carcinogenic and what is not, one could not reasonably expect the public to make such evaluations. The results from this study may, however, serve as a source of knowledge for public health and/or scholarly actors as well as health care professionals, forming a basis for providing evidence-based information on cancer risk factors, taking into account how common it is for people to endorse unestablished risk factors.

To get a deeper and fuller picture of the general public’s perceptions, we suggest that future studies should investigate these matters also from a qualitative perspective, focusing on beliefs regarding *how* these factors cause cancer, what risk management strategies are utilized, as well as investigating from what sources information has been gained. Regular follow-up of how these factors are perceived amongst the general public would also be a valuable contribution to the understanding of cancer risk factors awareness.

## Conclusions

A large proportion of the Swedish general public endorses a number of unestablished risk factors as carcinogenic. The heterogeneous awareness regarding different factors observed in this study illustrates how challenging it is for individuals to navigate complex and nuanced scientific jargon in a field of research where knowledge is constantly evolving. Consequently, the scientific community and public health authorities who communicate cancer risk factors, must face the complex and important task of ensuring that their messages to the general public are clear, transparent and correct.

## Data Availability

The datasets used and analysed in the current study are available from the corresponding author (EB), upon reasonable request.

## Abbreviations

AI: Artificial intelligence
AOR: Adjusted odds ratio
ECAC: European code against cancer
HCR: Health care region
IARC: International Agency for Research on Cancer
PAH: Polycyclic Aromatic Hydrocarbon
SD: Standard deviation
SEK: Swedish Krona
UPF: Ultra-processed food
WCRF: World Cancer Research Fund
